# The Luminescence Hypothesis of Olfaction

**DOI:** 10.3390/s23031333

**Published:** 2023-01-25

**Authors:** Kenneth Willeford

**Affiliations:** Coastal Carolinas Integrated Medicine, 10 Doctors Circle, STE 2, Supply, NC 28462, USA; willefordmd@yahoo.com

**Keywords:** olfaction, odorant, tunneling, luminescence, phonon, photon

## Abstract

A new hypothesis for the mechanism of olfaction is presented. It begins with an odorant molecule binding to an olfactory receptor. This is followed by the quantum biology event of inelastic electron tunneling as has been suggested with both the vibration and swipe card theories. It is novel in that it is not concerned with the possible effects of the tunneled electrons as has been discussed with the previous theories. Instead, the high energy state of the odorant molecule in the receptor following inelastic electron tunneling is considered. The hypothesis is that, as the high energy state decays, there is fluorescence luminescence with radiative emission of multiple photons. These photons pass through the supporting sustentacular cells and activate a set of olfactory neurons in near-simultaneous timing, which provides the temporal basis for the brain to interpret the required complex combinatorial coding as an odor. The Luminescence Hypothesis of Olfaction is the first to present the necessity of or mechanism for a 1:3 correspondence of odorant molecule to olfactory nerve activations. The mechanism provides for a consistent and reproducible time-based activation of sets of olfactory nerves correlated to an odor. The hypothesis has a biological precedent: an energy feasibility assessment is included, explaining the anosmia seen with COVID-19, and can be confirmed with existing laboratory techniques.

## 1. Introduction

The mechanism of the sense of smell remains unexplained. We know the anatomic location of the sense of smell occurs in the tissue of the olfactory epithelium (OE) which is an area of about 2.5 cm^2^ located in the superior aspect of the nasal cavity. The process of olfaction begins with the binding of odorant molecules to olfactory receptors (ORs). The corresponding olfactory nerves (ONs) then utilize a Gprotein-coupled receptor (GPCR) signal transduction pathway for activation [1]. The glomerulus is contained in the olfactory bulb of the brain and communicates with the olfactory cortex which interprets afferent information into olfaction [2]. Each ON expresses a single type of OR [3], and there are about 10^6^ receptor molecules on each of the cilia of ONs [4]. There is a high density of OR cells totaling 50 million in the OE [5].

**Figure 1:**
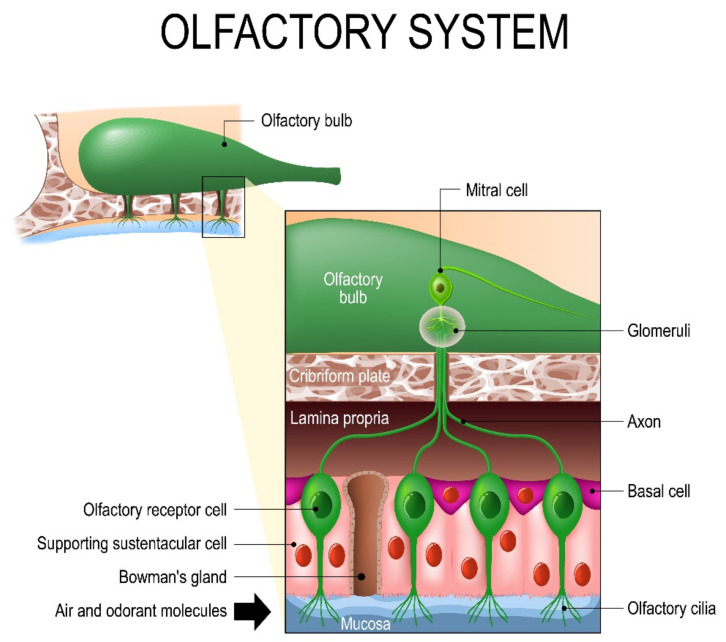
Olfactory epithelium with sustentacular supporting cells.

The morphology of human OE has been studied using scanning electron microscopy technology [6]. This has determined that the OE consists of four cell types, including basal cells, supporting sustentacular cells, ciliated olfactory receptors, and microvillar cells [7]. This structure is represented in Figure 1.

## 2. Shape Model

There are essentially only two groups of theories for the mechanism of olfaction and all of these as currently developed and understood theories lack either conceptual agreement or experimental confirmation. Early attempts to understand olfaction relied entirely on the odorant’s molecular shape alone, often referred to as a “lock and key” mechanism [8]. Here, a ligand binds to a receptor and initiates a response. This mechanism of receptor activation is well understood and is certainly true with many types of receptors. However, this is certainly not the only mechanism of olfaction. Contradictory evidence includes systematic studies showing shape alone as a poor predictor of odor [9]. The inadequacies of the shape model have been reviewed and include examples of striking exceptions to shape theory such as ferrocene and nickelocene processing a significant smell difference despite having very similar shapes [10].

## 3. Vibration Model

An alternate theory of olfaction is the vibration theory. This was originally discussed as The Scientific Basis of Odour in 1938 [11]. This was further developed and formalized as Odor and molecular vibration: neural coding of olfactory information in 1977 [12]. The landmark contribution to this theory came in 1996 with considerations of how the fact that deuterated analogs smell different cannot be accounted for based on structure-based theories, and for the first time, presented a mechanism for the vibration theory [13].

**Figure 2:**
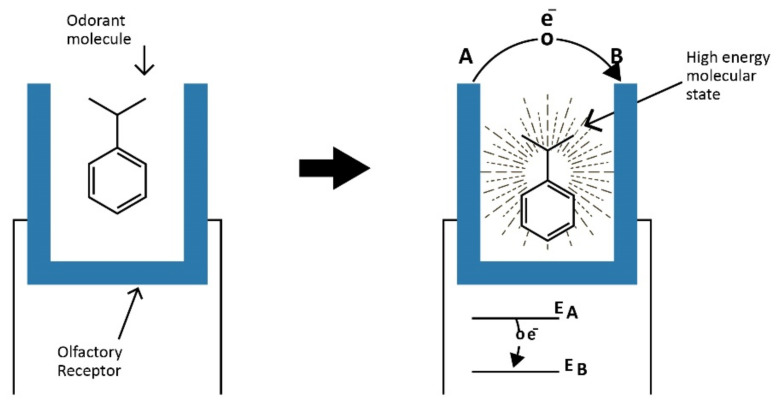
The inelastic electron tunneling mechanism is illustrated for human olfaction. When an odorant molecule binds in the receptor (**left**), an electron transition from a high energy state A to a lower energy state B occurs with the difference in energy creating a high energy molecular state of the odorant molecule (**right**).

This mechanism is based onthe quantum mechanics of physics and is the event of inelastic electron tunneling (IET). Quantum events occur on physical scales of nanometers. The activation of an ON from the OR is feasible in this regard because the dimensions of the ORs have been verified to exist on the nanometer scale [14]. The sequence of events is that an odorant molecule first binds to an OR. This is followed by IET where an electron within the OR “tunnels” to another location within the OR. The donor electron is initially at a higher energy state than it exists in after the IET event. The term ‘tunneling’ is used because there is no direct physical path for the electron to move from the first to the subsequent location. Since the electron cannot physically move to the new lower energy location it is said to have “tunneled” to this location. This phenomenon is based on particle–wave duality where electrons exhibit behavior both as a particle and as a wave. Here, there is a wave function in time and space where at a given time there is a wave function thatdefines probabilities of location in space. In physics, processes with no loss of energy are defined as elastic, and inelastic processes have a difference in energy of the initial and subsequent condition. In IET of the vibrational model of olfaction, as a result of the conservation of energy, when the electron tunnels to a position with lower energy, the difference in energy is imparted to the bound odorant molecule; this is an inelastic process, and therefore, this is IET Figure 2.

The next event in the vibrational model is that the tunneled electrons then flow through the OR protein and reduce a disulfide bridge via a zinc ion, and this releases the Gprotein of the GPCR signal transduction pathway for activation [13]. This signal transduction cascade then leads to the opening of ion channels generating a current which depolarizes the membrane of the ON [14].

An excellent example that supports the vibrational model is the similarity of the sulfurous smell of the vastly different structures of hydrogen sulfide with molecular formula H_2_S and decaborane with molecular formula B_10_H_14_. It is hypothesized that they both activate at least one receptor in common, supported by the fact they share similar vibrations at around 2600 cm^−1^ [10]. A second example supporting the vibrational mechanism is that odor character differences between deuterated and undeuterated odorant isotopomers with identical ground-state conformations, but different vibrational modes can be distinguished [15].

Many subsequent evaluations of the vibrational model have been largely in the field of physics rather than biology. The physical viability has been discussed with the reasoning that the donor electron must come from an amino acid of the receptor and was evaluated using calculated values for the Huang–Rhys factors of electron–phonon coupling for vibrational modes [16]. It is known that the operating principle of IET requires a linear geometry of the locations on the OR [17]. The energy transitions between particular OR protein residues comprising the walls of the OR have been studied [18]. A numerical analysis of the dissipative odorant-mediated inelastic electron tunneling mechanism of olfaction has been completed [19]. A fully quantum numerical model of the vibration-coupled IETS mechanism has also been presented [20]. Atomistic simulations yielding the Eigen-Value vibrational pseudo-spectrahas been presented and claimed to establish a physical basis for vibration-based odor classification, which harmonizes the Shape and Vibration theories [21].

However, there has never been a consensus opinion of the viability of the vibrational theory, and there have been skeptics. There was a relative paucity of research supporting the vibrational theory following the declaration in The Proceedings of the National Academy of Sciences that “there is no experimental data at the molecular level showing direct evidence of electron transfer—or the effect of odorant vibrations—being responsible for triggering odorant receptor response” [22]. Further research with GPCRs has questioned the viability of the IET mechanism through research with non-OR GPCRs, and concluded that if true, it would necessitate invoking exceptionalism of ORs within the GPCR class of proteins [23]. 

## 4. Swipe Card Model

As a result of the problems and inadequacies of both the shape and vibrational models, there has been an attempt to combine features of each of these in the swipe card model. The swipe card model includes the shape model of ligand binding to an olfactory receptor but adds that, following this event, additional information is conveyed by molecular vibration of the odorant molecule in the OR and uses an analogy that this is similar to the information encoded in magnetic swipe cards such as credit cards and hotel room keys. Inherent in the discussion of the swipe card model is the supporting and contradictory evidence of the shape and vibrational models with investigations of olfaction with molecular configurations of enantiomers and isotopes, as well as chiral and deuterated molecules. 

In the presentation of this model, it was noted that IET is well documented in inorganic systems and organic molecules such as cytochrome c but was new in signaling with the vibrational model. Conformational mobility is cited as a limiting factor in the vibrational model. A discussion was included with the time constraints of IET that olfaction occurs over milliseconds, which is significantly slower compared with most processes at the molecular scale. 

The swipe card model claims to provide a framework in which to evaluate critically the vibrational theory and to identify key questions including energy considerations and discusses several examples that challenge the role of inelastic electron tunneling in general. It was noted that progress in olfaction understanding is seriously limited by a lack of careful odorant physiological tests, and that much future mainly experimental research is needed. It concludes the vibrational model “cannot be the whole story” [10]. 

All three models of shape, vibration, and swipe have ligand to OR receptor binding leading to the GPCR signal activation of ONs. Each has been unable to completely explain olfaction. The Luminescence Hypothesis of Olfaction will now be presented, which is similar to the swipe card model in that it also includes both shapes with odor molecules binding to ORs and IET in the theory. It includes a new mechanism that, for the first time, explains how the brain can detect a multitude of odors, including the seemingly problematic simultaneously present mixtures, with a limited number of receptors. This hypothesis also explains the anosmia seen in COVID-19. Finally, this hypothesis makes predictions that can be tested and verified with existing technology and laboratory procedures. 

## 5. The Luminescence Hypothesis of Olfaction

This hypothesis begins with a discussion of the mathematical problems of other models. The number of types of ORs has been reported as 390 [24] and as more than 400 [25], in broad agreement with the number of functional genes, which have been estimated at approximately 400 [26], and the total of 339 intact OR genes which have been identified in the human genome database [27]. However, the number of odor molecules has been estimated as ranging from 400,000 to 1 million [28]. In addition, mixtures of different odorants can be detected allowing the distinction of more than one trillion olfactory stimuli [29]. Using the low number estimate for the number of odorants of 400,000 and the high number of receptors of 400, each receptor must be able to bind, on average, at least 1000 different types of odorants. 

There is general agreement that the brain must interpret an odor based on complex combinatorial patterns where each odorant must stimulate multiple ORs [30]. This is confirmed experimentally using a combination of calcium imaging and single-cell RT-PCR where each OR recognizes multiple odorants and each odorant is recognized by multiple ORs. In addition, different odorants are recognized by different combinations of ORs. [31]. Further studies of the olfactory bulb have also revealed a necessary time-based component to this complex spatiotemporal activity [32].

The number of possible combinations without repetition can be calculated using Equation (1):

Number of possible combinations without repetition = n!/[k!(n − k)!](1)

where k is the number of elements chosen and n is the number of elements to choose from.

The result of this calculation is that, if an odorant stimulates 2 of 400 ORs, there would be 79,800 unique combinations, and if an odorant stimulates 3 of 400 ORs, there would be 10,586,800 unique combinations. Since there are between 400,000 and one million unique odorants, the vast majority of odorants would be expected to stimulate 3 ORs. Some may activate two ORs, and some more than three, but the activation of three ORs for each odorant would provide for more than a sufficient number of unique combinations. 

The concept of complex combinatorial coding has previously been discussed, but the number of ORs in this coding has not previously been calculated. This is straightforward and logical. The major difficulty is in understanding how the timing of three separate types of OR activations can be recognized by the brain for chemical identity. The shape, vibration, and swipe card models all have one odorant molecule activating one OR at a time. So, three different odorant molecules must activate the three different types of ORs for that odorant’s unique “set” in a time span that is recognizable by the brain in a consistent time-dependent manner. This is the problem that has not been elucidated. 

## 6. False Sets

Consider the condition of odorant mixtures present at the OE. As an initial example with three odorants present, the set of ORs for Odorants A, B, and C can be represented by numbering the 400 different ORs:
OdorantOR setA**1**, 16, 200B42, **68**, 310C29, **150**, 380D [not present]**1, 68, 150**

So, the brain will be receiving afferent information from these nine ORs, which represent the three odorants. We know the brain can process this information in parallel, and that for each of the nine types of ORs, many will be occupied with the many molecules present for each odorant. With 50 million total ORs in the OE with 400 types of ORs, there are125,000 ORs for each of the 400 types. With a low estimate of a 10% occupancy rate, this is 12,500 of each of the 9 OR types present in this mixture, which calculates to 112,500 ORs being activated in the briefest of time. The brain must recognize all of this as sets of the three odorants. 

A compounding problem is that, with nine ORs, there are combinations of ORs being activated that may represent an odorant that is not present. For instance, the OR 1 of odorant A, the OR 68 of odorant B, and the OR 150 of odorant C are all being activated in this time span. However, this set of 1, 68, and 150 may be the OR set of an odorant that is not present. We can calculate the number of combinations that are present that may be the set of an odorant that is not present. Using Equation (1), there are 84 unique combinations of three of nine possibilities. Three of these sets are present, but there are 81 other sets of three that could represent odorants that are not present. Many of these would be meaningless ‘noise’ since there are many more possible combinations of 3 of 400 than there are odorant types, but some would also represent odorants that are not there. Similar to auditory research where the brain must compare sounds to a lexicon to understand language, here the brain must compare these sets to a lexicon to experience chemical identity. In this example the brain is bombarded with 112,500 OR signals; some are the sets of the three odorants present, most are sets that do not correlate to anything, and some are the sets of odorants not there. This is with each of the 10% of occupied ORs only sending one signal, and we will see many, many more signals from each OR are required. 

This becomesmuch more complex when there are 30 molecules present, and we know the brain can distinguish 30 types of odorants simultaneously present [29]. Now using 90 ORs for the 30 sets of odorants and 10% occupancy, the number of activating ORs is:

(125,000 ORs for each of the 400 types)(90 types in this condition)(10%) = 1,125,000.

The problem is orders of magnitude more difficult than this because this one million, one hundred twenty-five thousand is the number of activating ORs the brain would analyze to determine that there are 30 types of odorants present. The brain simultaneously must determine that other combinations of OR activations are meaningless. Again using Equation (1), there are 117,480 unique combinations of 3 of 90. Thirty of these represent the odorants that are there, and there are another 117,390 sets of three that represent either nothing or odorants that are not there. 

Yet, there is still another level of complexity that is within the brain’s ability. The brain can distinguish mixtures of odorants thatare simultaneously present. Experimental results show that humans can discriminate the “astonishingly large number” of 1.72 × 10^12^ mixtures of 30 components out of a collection of 128 odorous molecules [29]. These mixtures are created with differing concentrations of the 30 components. Somehow the brain can distinguish trillions of mixtures. Even with 100% occupancy of the 125,000 ORs of each of 90 types, this is 11,250,000. This 11 million is five orders of magnitude less than the 1.7 trillion detectable mixtures. Clearly, many ORs are activated many times in this brief period of time to present information to the brain for the interpretation of concentrations and ratios in addition to identifying 30 odorant types. This seems an unsolvable problem even without considering the other 117,390 sets of three that represent either nothing or odorants that are not there. Olfaction sensory processing would be expected to operate on ms timescales as we know is true with auditory and visual stimuli; however, with these numbers, the sensory information being sent from the OE must be at rate orders of magnitude quicker. Incidentally, the quantity of molecules is not felt to be a problem since a single mist droplet contains up to millions of molecules and a single drop of water contains over one sextillion (1.0 × 10^21^) molecules, and any one odorant molecule may dissociate from one OR and immediately activate others. 

The next consideration is the 1:1 correspondence of an odorant molecule to OR activation, as is presented in each of the shape, vibration, and swipe card models. Here, different portions of each of the three individual molecules of the same odorant must be bound by the three different ORs of a set. This must be true for each of the 400,000 to one million odorants where different portions of the structure are bound to 3 of the 400 types of ORs. We know that some molecules do have binding to more than one receptor but that all 400,000 do is pure speculation. We have calculated that, with the low estimate of 400,000 odorants and 400 OR types, each OR binds, on average, 1000 types of odorants. Since each odorant set is three, now each OR must bind, on average, 3000 types of odorants. With 1:1 correspondence, the ORs must be nonspecific enough so that each can bind 3000 types of odorants. However, they must be specific enough so that each of the at least 400,000 odorants binds to a unique set of three. The chances that this is true reach the highest levels of improbability.

How the brain knows an odorant is not there when each of the sets of threeisbeing activated in the same time span as those OR sets of odorants that are present has not previously been considered or discussed. There are far more of these “false sets” than real sets. In the example with 30 odorants, there are 117,390 “false sets” compared to the actual real 90 sets. That’s an astounding 99.9% that are “false sets”. It is in this context that the greater than one trillion mixtures are distinguished. Further, while the ability of the brain to distinguish 30 different odorants simultaneously present has been confirmed, the limit of the number of simultaneously present molecules which can be distinguished has not been evaluated and may be greater than 30, making all of this even worse for proponents of a 1:1 correspondence. With the number of molecules present in these mixtures, there are potentially billions of molecules activating millions of nerves that the brain is interpreting as groups of three for chemical identity. How all of this occurs in split seconds is astronomically incomprehensible. It is a critically important factor that makes a 1:1 correspondence exceedingly improbable because we know the brain can tell without error what is there and in what proportions, and also what is not there.

## 7. Timing with 1:1 Correspondence

The problem is that, with each molecule activating one OR, then the timing of the activation of the individual ORs of the set of three is random. The next time any of the odorants are present in the OE, the timing of the activation of the individual ORs of the set of three would be different. This timing of the binding of three separate molecules of one odorant to the corresponding three ORs would be influenced by many factors, including respiratory rate and depth, since this is the process that brings the odorant molecules in proximity to the ORs for binding. There are expected to be variations in airflow patterns and perhaps eddy currents also present at the OE. Changes in concentration would clearly create differences in the timing for the three bindings with higher concentrations expected to create decreased time between the three OR activations. Temperature and other environmental conditions may also be factors. For at least these reasons, the interval timing of OR activations are random events and there cannot be consistent time intervals between the bindings of the three individual odorant molecules and the corresponding ORs of the set. Without consistent interval timing, there appears to be no conceivable way the brain could interpret this information as the sense of smell. The brain cannot recognize a pattern when there is no consistent pattern. This is an unsolvable problem with a 1:1 correspondence of one odorant molecule activating one OR, and this is the presented mechanism of the shape, vibration, and swipe card models.

This assertion that the precise timing intervals between the three OR activations of an odorant set are random over time is a pivotal tenet of The Luminescence Hypothesis of Olfaction. Returning to the environment at the OE with 30 simultaneously present odorants, it has been presented that the brain can distinguish trillions of mixtures interpreting the afferent information from 30 sets of three ORs with a total number of ORs of up to 11,250,000, which is diminished only by the receptor occupancy rate. With trillions being so much larger than 11 million, each OR must be activated a multitude of times in just a split second. The brain would need to distinguish this among the overwhelming odds of 99.9% or 117,390 “false sets” to the 90 true sets of odorants actually present. With this number of events occurring in such a brief period, the timing intervals between the three OR activations of an odorant set must be of a level of precision and reproducibility that far exceeds the binding of three separate molecules of the same odorant to three separate ORs in the context of all the confounding variables that affect any individual molecular OR binding. This detailed mathematical description defies an explanation utilizing a 1:1 correspondence.

Three separate molecules of the same odorant binding to three separate OR’s can be logically excluded based on the randomness of the timing of these multiple bindings in the context that they must be interpreted by the brain as a set, with a corresponding time restraint which must be consistent for that odorant. It would be expected that the timing must be “near simultaneous” because likely tens of thousands of other molecules of other odorants are simultaneously present and must also activate a set of three ORs, and the brain is able to distinguish mixtures. 

This condition of OR sets of three necessitates a mechanism of 1:3 correspondence where a single molecule elicits the response of three different ORs in such a brief period of time that a different odorant, with a different and unique set of three ORs that is simultaneously present in the OE, can also be distinguished by the brain. 

This complex combinatorial coding with consistently reproducible time dependence is well explained with an analogy to music and hearing. Music has twelve notes in an octave including the flats and sharps. A chord is defined by three or more unique notes sounded together. Three unique notes simultaneously experienced by the ear areinterpreted by the brain as a cord. If the three notes are separated in time they are not perceived as a cord, and if repeated attempts have inconsistent timing between the three notes it will just result in random incomprehensible noise. This becomes worse if more than one cord is attempted, each with three unique notes and all separated with inconsistent intervals of time. It is the combination of a unique set of three notes experienced simultaneously that creates music. In very much the same way, three ORs experiencing activation together in time is interpreted by the brain as an odor. 

## 8. Mechanism for a 1:3 Correspondence

The Luminescence Hypothesis of Olfaction provides a mechanism for greater than or equal to a 1:3 correspondence where a single odorant molecule activates multipledifferent ORs in “near simultaneous” time such that the brain interprets this activity consistently with a corresponding sense of odor. The Luminescence Hypothesis of Olfaction has a classical end to a quantum beginning. The first event is the binding of an odorant molecule to an OR. This is followed by the quantum event of IET leaving the odorant molecule in an excited state Figure 2 [20,23]. Previous discussions of IET in regard to olfaction have focused on the tunneled electron and possible Gprotein-mediated signal transduction pathway ON activation. In this model, the location and destiny of the tunneled electron arenot important. It is the high energy state of the odorant molecule thatleads to the “near simultaneous” activation of three ONs.

Luminescence is the emission of light from an excited electronic state of a molecular species. There are fluorescence or phosphorescence luminescence bands which are differentiated based on the average lifetime of the excited state, where phosphorescence is much longer than fluorescence. The stored energy of an excited electronic state is dissipated radiatively with the emission of photons which is luminescence, or non-radiatively with the release of thermal energy. There are two mechanisms for non-radiative energy transfer processes, which are the Förster resonance mechanism and the Dexter exchange mechanism. The Förster resonance mechanism functions with donor–acceptor distances as long as 50–100 angstroms, and the Dexter exchange mechanism involves direct contact between the donor and the acceptor atoms or molecules [33]. Each of these modes of energy dispersion of the high energy state of the odorant molecule following IET will now be considered in the context of providing for the required greater than or equal to 1:3 correspondence.

Fluorescence results from the molecular transition from the singlet state to the ground state and has a typical lifetime of nanoseconds. Phosphorescence results from the molecular transition from the triplet state to the ground state and has a typical lifetime of milliseconds. Organic molecules only showing efficient fluorescence and phosphorescence are extremely weak. This is because the transition from the triplet state to the ground state with phosphorescence is quantum mechanically forbidden, requiring the participating electron to undergo a spin flip. The triplet state in organic molecules is typically considered a “dark” state because there is nonradiative recombination at room temperature [34]. This excludes phosphorescence.

Considering the required “near simultaneous” activation of three ONs with non-radiative energy transfer, the Dexter exchange mechanism is excluded since there is no continuity between ORs which are physically separated by sustentacular cells. The Förster resonance mechanism of non-radiative energy transfer is also excluded. This exclusion is based on the distance where the proximity of ORs for the Förster resonance mechanism of non-radiative energy transfer to operate would require distances of 50–100 angstroms between ORs, and the actual distance is calculated to be on the order of a few micrometers based on 50 million ORs contained in the 2.5 square centimeters of the OE. An exhaustive analysis of all possible modes of energy dispersion of the high energy state of the odorant molecule following IET would include thermal transfer to the receptor to which the odorant molecule is bound. This is excluded in this hypothesis because this cannot provide for the required greater than or equal to 1:3 correspondence.

The odorant molecule following IET can develop a high energy state by the excitation of electrons from lower energy states to higher energy states or by excitation of vibrations from lower amplitude oscillations to higher amplitude oscillations. This is evidenced by both vibrational and electronic IET spectroscopy acquisitions from single molecules [35]. The previous vibration and swipe card models considered only vibrational excitations. The Luminescence Hypothesis of Olfaction differs from these in that it considers both modes of high energy. Electronic excitation is considered more likely to create the required three photons since this mode is of much higher energy than vibrational excitations.

## 9. All Things Considered

A set of three ORs per odorant type is a mathematical certainty. The 3:1 correspondence with the release of three photons from the high energy state of an odorant molecule following IET has been discussed. In this situation, the OR that binds the molecule may not be among the set of three. 

Another scenario is that the OR that binds the molecule is among the set of three, and in this case, the release of two photons would meet the 3:1 correspondence. This would be one OR/ON activation from classical ligand binding to a receptor, coupled with two OR/ON activations with quantum IET. Incidentally, these two photons must be of different energies, so a quantum entangled pair would be excluded.

An additional consideration is that there exists a 1:1 correspondence with individual odorant molecules activating individual ORs and that three separate molecules are activating the corresponding three OR set in a numerically non-proportional manner. In this scenario, the three types of ORs corresponding to an odor experience different rates of excitation, and the brain would need to interpret this differential proportionality of receptor types activation as odor identification. However, this nuance of the 1:1 correspondence models does not solve the false set problem or the presented problems with reproducible interval timing of the components of the sets to be identified as sets. The fact that the brain can recognize with 100% accuracy sets of three ORs when there are over 1 million ON activations in split-second timing when the actual true sets representing chemicals identify is only 0.1% of all of the combinations is an unresolved challenge for any model with a 1:1 correspondence. 

## 10. Can IET Produce Photons?

The key question is if IET can produce photons. The answer is yes, and for both types of excitation states and for both inorganic and organic molecules.

Light emission by IET has been known for many years [36] and can be from single or from higher-order multielectron inelastic tunneling [37]. The phenomenon of light emission via the inelastic tunneling of electrons through potential barriers or junctions has been used in the development of micro- or nanosized electrically controlled sources of optical radiation for use in optoelectronic systems [38] to develop miniaturized light sources and ultradense photonic instruments in the visible and near-infrared spectrum [39] in research and development of optical antennas [40] and scanning tunneling microscopy. Scanning tunneling microscopy photon emission through IET is known as scanning tunneling microscopy-induced luminescence and is useful for the study of the intrinsic luminescence of molecules in their chemical environments as well as allows charge transfer to be monitored with submolecular resolution [41].

A common method utilized to electrically excite photons via in elastic electron tunneling is metal–insulator–metal tunnel junctions [42]. Noble-metal films can also exhibit electroluminescence through inelastic electron tunneling [43]. The optical properties of IET tunnel junctions have been controlled by changing the shape or the material of the electrodes with the aim to improve photon emission efficiencies [44]. In addition, the resonant peak of emitted photons from IET can be spectrally tuned [45]. Interestingly, electroluminescence following IET has been found to be linearly polarized [43].

Organic molecules are also confirmed to exhibit photon emissions following IET. For example, organic molecules have been utilized in the junction of scanning tunneling microscopy-induced luminescence [46]. Photon emissions from organic polycyclic aromatic hydrocarbon molecules following IET have also been investigated [47]. Photon emissions following IET in organic molecules are not solely from excited electronic states as evidenced by the molecule porphyrin, which exhibits intrinsic fluorescence and produces photons from molecular vibronic transitions [48]. 

Significantly to The Luminescence Hypothesis of Olfaction, research in quantum computing has revealed two or more photons emitted from a single molecule as a result of IET. The production of multiple photons from a single molecule is termed bunched emission, and the time between the releases of the photons in bunched emission following IET has been measured to be less than nanoseconds and in the range of 50 picoseconds [49].

**Figure 3:**
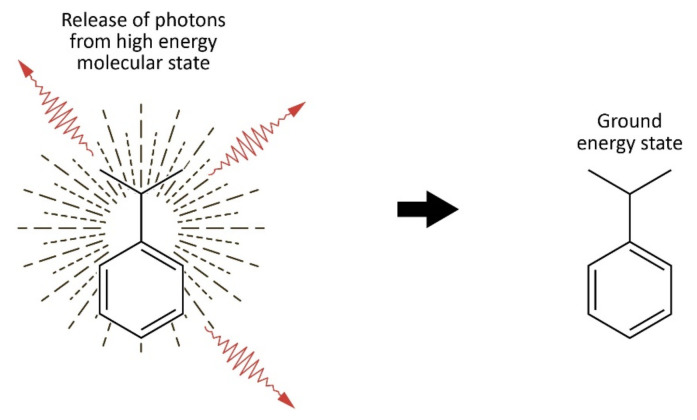
Odorant molecule in high energy state following IET (**left**). Emission of three photons as the odorant molecule decays to the ground energy state (**right**).

Of the four types of luminescence decay, only fluorescence is possible with this proposed mechanism. The Luminescence Hypothesis of Olfaction involves fluorescence luminescence with radiative emission of multiple photons from an excited molecular state of odorants in the ORs which results from IET Figure 3.

**Figure 4:**
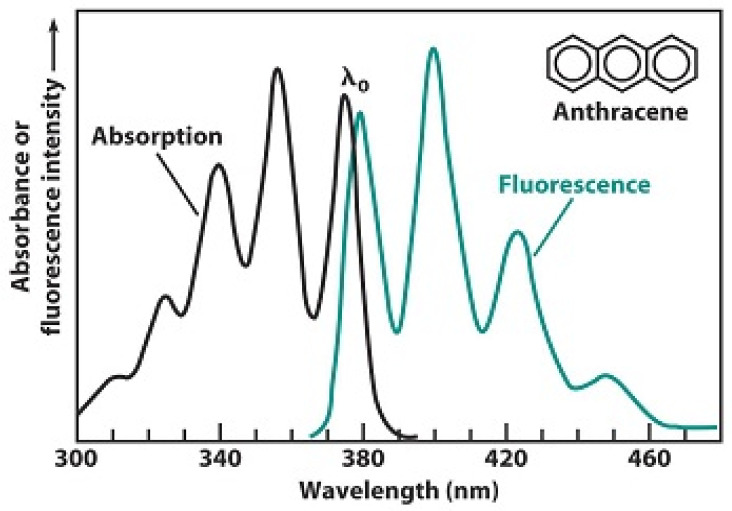
Excitation and emission spectra of anthracene that have the same mirror image relation at the absorption and emission spectra. Adapted from Byron, C.M. and Werner, T.C., *J. Chem. Ed.*, 1991, 68, 433.

The next consideration is the number and energy of the photons which would be released from odorant molecules thatdecay from a high energy state. Each odorant has a unique molecular structure that has unique resonances when in an elevated electronic energy state. The energy of these resonances corresponds to the high energy state of the molecular bonds. The molecular bonds of odorant molecules have been studied with excitation by absorbing infrared light with specific wavelengths [26]. Very much like IR spectroscopy reveals absorption wavelengths of organic molecules, the emission spectra of luminescence reveal the emission wavelengths. They are similar in that they both are unique and based on the molecular bonds of the organic molecule. The emission spectra areat lower energy than the absorption spectra because the absorption of energy and the subsequent emission is not 100% efficient. Wavelength is related to energy by E = hc/λ. As an example, Figure 4 represents the excitation and emission spectra of anthracene. There are three strong and one weak emission bands from the decay of the high energy state of anthracene. Similarly, high energy states of molecular vibration modes have unique decay energies.

Figure 4 depicts the high energy state being achieved through IR absorption; in the Luminescence Hypothesis of Olfaction the high energy state results from IET, and a similar emission spectrum would result. An experimental database of the optical properties of organic compounds has been created and includes emission wavelengths [50]. In a separate report, 11,460 experimentallysynthesized fluorescent organic molecules were extracted from the Reaxys database with corresponding emission wavelengths between 200 and 900 nm [51]. This mechanism creating an emission spectrum can provide for the required 1:3 correspondence where a single odorant molecule results in three photons. 

## 11. Activation of ORs by Photons

The next event in The Luminescence Hypothesis of Olfaction is the activation of ORs by photons. This has never been suggested but is not without precedent. Rhodopsin is the photoreceptor of rod cells in the retina and absorbs light to mediate the first step of vision by activating the G protein transducin. Rhodopsin’s light-sensitive ligand 11-cis-retinal is covalently bound to the apoprotein via a protonated Schiff base. Light absorption induces 11-cisto all-transisomerization and begins a cascade of events in the G protein signal transduction pathway [52]. Very much like light travels through the cornea and vitreous to the retina, in the Luminescence Hypothesis of Olfaction light travels through sustentacular cells to ORs. 

The next consideration is the structure of ORs. The A–F system of GPCR classification identifies six classes based on the amino acid sequences and functional similarities. The OR is designated in Class A, which is known as the “rhodopsin-like family”, the same family as the light-sensitive photoreceptor of rod cells in the retina [53]. Within this family, the OR subfamily is the largest of Gprotein-coupled receptor families [54]. The common features and distinct characteristics of sensory transduction in photoreceptors and ONs have been evaluated. This includes a discussion of signal detection, cell morphology, electrophysiology, sensitivity, detection of stimuli, discrimination between stimuli, sensory transduction activation, amplification, receptor and Gprotein inactivation, and adaptation. ONs are described as sharing “an amazing level of similarity” to photoreceptors in several aspects. In addition, the OR complexity compared to photoreceptors is evidenced by the use of five opsin genes to cover the visible spectrum, whereas ONs use hundreds of OR genes to cover the odor space [55]. The isolation of genes that code for olfactory receptors which showed they belonged to the class of Gprotein-coupled receptors led to the award of the 2004 Nobel Prize in Physiology or Medicine [56], exemplifying the importance of olfaction research in the scientific community. 

The structure of other G protein-coupled receptor proteins is characterized by seven hydrophobic membrane-spanning domains. The ligand binding of ORs has been described “to resemble rhodopsin”. However, the conformational changes of the OR with interactions with odorant molecules are not fully understood [57]. ORs have been reported to have “structural mimicry” with rhodopsin, and the mechanism of signaling activation through a conformational change in the transmembrane segments is believed to lead to interactions with Gproteins [58].

However, there remains an incomplete understanding of both the structure and function of ORs. For instance, the structural changes ORs undergo at the molecular level areundetermined [1]. In fact, there is a total absence of an experimentally verified structure for ORs, and the quest has been called “elusive”. The reasons include experimental difficulties not encountered in research of other types of receptors. One of these is that ORs are not efficiently transported to the cell surface when expressed in cells other than olfactory sensory neurons [59]. There appears to be something special and mysterious about ORs. What is known seems to support the tenets of The Luminescence Hypothesis of Olfaction, and what is unknown leaves open the real possibility that it is true. 

## 12. Luminescence Timing

The Luminescence Hypothesis of Olfaction is dependent upon the consistent timing of the activation of each of the ONs associated with the unique set of ORs for a particular odorant molecule. This required consistency has been presented based on the brain’s ability to interpret all situations with odorants present at the OE as an experience of a particular odor. The decay of the high energy state of an odorant molecule in an OR following IET leading to multiple photons is the beginning of this timing assessment. For this hypothesis to be true, it would require these photons to be emitted in such a brief period of time that they are cumulatively recognized by the brain as a set. This has been experimentally measured in picoseconds [Leon]. This timing is the activation of the set of three ORs, distinct from the known ms processing time of the brain. Because these will travel literally at the speed of light, and the distances to adjacent ORs of the set contained within the OE are so small, the time from the release of a photon to arrival at the other ORs is not a confounding variable. 

There is extensive research that supports this required timing condition of The Luminescence Hypothesis of Olfaction. The event of IET occurs on the order of nanoseconds [10]. Chemical sensing is one of the explicit purposes that fluorescence lifetime standards have been developed. These standards reveal that fluorescence lifetimes vary from picoseconds (ps) to nanoseconds (ns), and significantly, the decay times are independent of the excitation and emission wavelengths [60]. Subsequent single photon timing spectroscopic techniques have confirmed ps time constants [61]. Extended fluorescence lifetimes would be beneficial in certain applications and the cloning of green fluorescent proteins has been accomplished including mutants, but lifetimes have not been able to be extended beyond the ns range [62]. The time intervals of ns and ps are found in both organic and inorganic molecules and discussions have included both orbitals and bonds as sources [63,64,65]. The timing on this scale appears to be universally true and ps have also been measured with chlorophyll fluorescence decays [66]. Verification of ns and ps times are based on the experimental techniques of single-molecule spectroscopy, diffuse optical tomography, time-resolved emission spectra, and advanced time-correlated single photon counting relying on multidetector and multidimensional acquisition [67]. 

The “dark state” in fluorescence, which is the unsupported concept that there is a time lag between the moment of excitation and the beginning of emission is believed to be very short, even shorter than the emission, and has been called “nonexistent”,with evidence that “remain scarce” [65,68]. All of these times are orders of magnitude shorter that the dwell time of the odorant ligands on the OR which are on a millisecond timescale [69].The combination of a brief, if even existent “dark state”, along with ns and ps fluorescence lifetimes indicates the release of the multiple photons of an odorant set resulting from the decay of high energy states of odorant molecules in ORs following IET would be on a time-scale so brief that they would be experienced as a set by the brain, providing for the time basis for the interpretation of sets of ONs of individual odorant molecules.

## 13. Overlap

An analysis of the number of molecules present at the OE with a mixture of 30 odorants, the corresponding 90 types of ORs, the mathematical certainty of sets of three for chemical identity, and false sets leads to the concept of overlap. A logical explanation of how the brain is able to distinguish real odor sets from false sets is that there is an interval of time where only true sets are experienced. This window of time must be very brief since there are over one million ON activations in the condition of 30 mixtures in split-second timing. Real OR sets being activated in a time span without overlap of other sets is considered. If there is an overlap in time of OR activations from differing sets it would seem incomprehensible that the brain could sort that out. This set time interval can be estimated. With a mixture of 30 odorants and the corresponding 90 OR types, a total of 50 million OR cells at the OE, a sensing time of a half second, and a low estimate that each OR is activated only once, the set time interval is calculated as:

set time interval = (0.5 s)/[(90/400 OR’s)(50 million)(1 set/3 OR’s)] = 130 ns/set.

The sensing time is likely less than one half second and since the speed of biological systems is so high; a realistic estimated set time interval would be a factor of ten less or 13 ns. This is the time span that a set of three ORs can be activated without overlap from other set activations. This calculation estimates the maximum interval without overlap; perhaps it is much less, even on the order luminescence emission times.

Considering the multitude of molecules present at the OE with a mixture of 30 odorants, it would seem to be impossible with a 1:1 correspondence that the individual bindings of three separate molecules of an odorant to three ORs could occur without overlap in ON activation timing with the bindings of other odorant molecules from the other 29 odorants present. If there is overlap, as would be expected with a 1:1 correspondence, false sets would result. 

However, the luminescence timing of picoseconds to nanoseconds does fit well with this analysis. This is sufficiently fast so there would not be overlap in OR activations in a set time interval, leading to set identification by the brain and perceived chemical identity. 

## 14. Signal Processing

This is essentially a pattern recognition problem, the brain must be able to recognize the pattern or set of three OR’s each and every time an odorant molecule is present at the OE in any and all conditions. This molecular recognition stage of olfaction is integrally associated with how the brain interprets the afferent information from OR sets, and insight may be gained from previous research which has established the brain processing time of milliseconds for both vision and hearing. 

Investigations of vision have revealed that the brain processing time is hundreds of milliseconds, and also that our eyes typically fixate on an object for hundreds of milliseconds. This means information reaching our eyes at different moments is processed in the brain together [70]. We also know that single neurons in the human auditory core fire as early as 10–20 ms in latency following an auditory stimulus, but that the first stages of neuronal processing in the primary auditory area peaks around 50ms after sound onset, meaning that first stages of neuronal processing in the neocortical circuit occur in about 30 ms [71]. Further, properties of a speech sound are neurally encoded for more than 500 ms after the speech sound has dissipated from the acoustic signal [72]. This research utilizes the perception of phonemes which are distinct units of sound that distinguish one word from another, such as the pronunciation of the letters p,b,d, and tin the words pad, pat, bad, and bat. These time intervals mean that the brain processes multiple phonemes at the same time. In addition, the phonetic representations of the three most recently heard phonemes are maintained in parallel, and the activity pattern encoding these features evolves, systematically, as a function of elapsed processing time, which prevents consecutive speech sounds from co-occupying the same activity pattern. The fact that speech sound properties are neurally represented for much longer than the sensory input permits the auditory system access to the history of multiple phonemes simultaneously. Finally, the processing trajectory for human speech processing does not trace a widespatial path across distinct regions, and instead, phonetic features remain locally encoded for up to 400 ms, then the trajectory to higher centers is different for different features, indicating there may not be a strict anatomical transposition from low to high-level areas [73].

A major difference in considering olfaction compared to vision and hearing is that, with both vision and hearing, the brain is encoding sequencing in a time-dependent manner in order to experience motion and speech. With olfaction, chemical identity is paramount. Perhaps the concentration of a single odorant can be interpreted by the brain as the rate of set activations, and the concentrations in mixtures are interpreted by the proportional ratio of sets. The ability of the brain to interpret complex signals is evidenced by vision and auditory research. What must be explained with olfaction is the details of what and how information is presented to the brain for the interpretation of chemical identity.

What needs to be considered here is that while the set of three OR activations is presented to occur as rapidly as picoseconds to become a “set” in time, can the much longer signal processing time of milliseconds allow the brain to interpret the afferent information for chemical identity? This is where the previous research in vision and hearing provides for the continued viability of The Luminescence Hypothesis of Olfaction in this regard. Results in both vision and hearing investigations have confirmed that in both cases sensory input occurs more rapidly than signal processing times and that multiple afferent vision and hearing stimuli are processed at the same time, maintained in parallel, and are neurally represented much longer than the sensory input. So, chemical identity with olfaction can be understood to be very similar to visual and auditory senses. The hypothesis is that sets of three are experienced so rapidly that they are separated in time sufficiently to be distinct from other sets of three from the many molecules present, and these are processed much slower, with the maintenance of set identity while other sets are experienced.

## 15. Energy

A final consideration in the viability of The Luminescence Hypothesis of Olfaction is energy. It is known that vision in humans occurs through the absorption of photons from 400 to 780 nm by photoreceptors in the retina. The mechanism involves acis, trans-geometric isomerization of the 11-cis-retinal chromophore found in rhodopsin in rods and also in cone opsins of retinal photoreceptors. These photons carry energy in the 2.5 eV range, but only 1.5 eV per opsin molecule is used to elicit protein conformational changes [74].

In The Luminescence Hypothesis of Olfaction, the energy contained within the high energy state of the odorant molecule following IET is released in the multiple photons of an odorant set. The important question here is if there is sufficient energy in this decay to create three photons, each with sufficient energy to elicit activation of an ON.

While the majority of vibrational theory proponents promote a single phonon process, multiphonon processes have also been discussed and have been determined to be as quick as the single phonon process, suggesting that contributions from different phonon modes of an odorant molecule should be included for electron transfer in olfaction. However, both models are limited by the energy difference between the donor and acceptor states in the olfactory receptor using the IET mechanism, which is widely reported as 200 meV [75]. However, this energy of 200 meV is not certain, could result in an electronic and/or a vibrational high energy state, and could be higher allowing for the emitted photons to be in the visible and/or IR range.

Since the full structure and function of ORs is unknown, direct comparisons to rhodopsin are not possible. Since the ORs are in the rhodopsin-like family, it is reasonable to consider the mechanism of activation to be similar to the retina’s response to photons. A practical strategy to approach this problem is to consider the mechanism with rhodopsin, which involves a cis-to-trans configuration conversion of an alkene. The energy requirements for this are established for many molecules. For instance, the cis–trans energy difference for the peptide bond in the gas phase and in an aqueous solution of N-methyl acetamide is 2.5 kcal per mole, which calculates to 0.11 eVper molecule [76]. The cis–trans isomerization of azobenzene is 2.9 kcal per mole [77], and the intramolecular competition between cis and trans formamides has been shown to be less than 1.0 kcal per mole [78]. It is believed that ORs use the GPCR signal transduction pathway for activation, but there may be an activation mechanism of lower energy than cis to trans isomerization. These values are presented for comparison based on the conjecture that ORs use the same mechanism of cis to trans isomerization, as is the case with rhodopsin, and if so, The Luminescence Hypothesis of Olfaction is energetically possible. 

## 16. COVID-19

Research on the mechanism of the total loss of smell, or anosmia seen in COVID-19, hasbeen performed with the hopes of gaining insight into the mechanism of olfaction. The reasoning is that if we can understand why it suddenly doesn’t work, maybe we can understand how it does work. However, at this time, the cellular and molecular mechanisms of anosmia with COVID-19 remain unclear [79]. A downregulation of OR genes has been detected [80], but downregulation could explain altered olfaction, not the total loss of the ability. COVID-19 has a high prevalence of anosmia of 38% [81]. The source has not been discovered to be related to the transmembrane serine protease TMPRSS2 which is involved with cell entry [82]. The main finding in multiple research efforts is that it is the sustentacular cells that mediate the dysfunction, and not the neural cells of ONs or the brain [80,83].

The Luminescence Hypothesis of Olfaction can explain the anosmia of COVID-19. We know the sustentacular cells are affected byCOVID-19 with inflammation and changes in density [80,83]. This would be expected to result in a change in the index of refraction of the sustentacular cells. This change in the index of refraction with the corresponding change in the velocity of photons through the sustentacular cells would disrupt the precise timing of any set of photons that the brain is interpreting as corresponding to an odor. In The Luminescence Hypothesis of Olfaction, the sustentacular cells are the communication medium that provides for the 1:3 correspondences which areinherently unique tothis hypothesis. A prediction of The Luminescence Hypothesis of Olfaction is that COVID-19 results in a change in the index of refraction of sustentacular cells, and that recovery of olfaction correlates with a return to the normal index of refraction of sustentacular cells. 

## 17. Validation

The developmental process for The Luminescence Hypothesis of Olfaction began with the calculation of the number of possible combinations without repetition because of the limited number of OR types of about 400 and the brain’s ability to distinguish more than 400,000 odors. It considers the previously unresolved problem of how the timing of ON activation from three separate molecules of the same odorant can be activated in a consistent time span to be experienced as a set for interpretation by the brain as an odor, in the context of other simultaneously present odorants, each which must also activate a different set of ONs. The condition where greater than a million ONs representing up to 90 OR types being activated by a multitude of individual molecules simultaneously present at the OE from 30 different odorant types was discussed in the context where up to 99.9% of sets of three are ‘false sets’ and all of this occurs in split-second timing. It is presented that this is a receptor-level recognition pattern and cannot be sorted out by higher-level processing alone. Possible factors cited that prevent consistent and reproducible binding to the OR sets for three separate odorant molecules included respiratory rate and depth, concentration, temperature, and other environmental conditions. It is this seemingly unsolvable problem that there mathematically must be sets of ONs to correlate to an odor, in the context they must arrive inthe brain with consistent timing intervals that preclude a 1:1 correspondence with one molecule to one OR. 

The Luminescence Hypothesis of Olfaction is the first to presentthe necessity of or mechanism for a 1:3 correspondence. The sequence of events has been explained as the binding of an odorant molecule to an OR, IET with an odorant molecule created in a high energy state, fluorescence luminescence decay with radiative emission of three or more photons for the majority of odors, near simultaneous activation of the multiple ONs of an odorant set, and finally, the transmission of this set of signals to the olfactory cortex of the brain for recognition of chemical identity. 

Discussions of timing and energy and a mechanism to explain the anosmia of COVID-19 may be interesting, even supportive, but ultimately the destiny of this theory lies in experimental validation. Fortunately, this is a relatively easy task for the olfaction research community. A good model is able to make predictions, and The Luminescence Hypothesis of Olfaction predicts a change in the index of refraction of sustentacular cells with COVID-19. This can be tested in a similar manner as the techniques used to determine the index of refraction of the cornea to be 1.376 [84]. The definitive test for The Luminescence Hypothesis of Olfaction will be through observations of ON activation with electromagnetic radiation in the energies of the luminescence of organic odorant molecules. Several detection methods have been used to quantify receptor activation in heterologous systems, including the secreted placental alkaline phosphatase assay, the luciferase assay, and real-time cAMP assays [85]. Real-time in vitro monitoring of odorant receptor activation by an odorant in the vapor phase has also been measured using cAMP release [86]. Of these, calcium imaging would appear to be an attractive initial experimental technique to determine the truth of The Luminescence Hypothesis of Olfaction. Experimental verification of OR/ONs responding to photons would be the initial validation and support. No other currently proposed mechanism would predict this, and the existence of photon reactive OR/ONs would lead to further investigations to test the 1:3 proposed correspondence. A further investigational plan would be experimenting with photon detectors to measure group photon emissions with time resolution sufficient to detect if sets of three photons are released from ORs on this time scale.

The Luminescence Hypothesis of Olfaction has multiplicity, reproducibility and consistent timing, is energetically possible, has a biological precedent with the retina, explains the anosmia of COVID, predicts a change in the index of refraction of sustentacular cells in normal vs. COVID-19, and can be validated with existing laboratory techniques.

## Data Availability

Not applicable.

## References

[1] Sharma A., Kumar R., Aier I., Semwal R., Tyagi P., Varadwaj P. (2019). Sense of Smell: Structural, Functional, Mechanistic Advancements and Challenges in Human Olfactory Research. Curr. Neuropharmacol..

[2] Welge-Lüssen A. (2005). Re-establishment of olfactory and taste functions. GMS Curr. Top. Otorhinolaryngol. Head Neck Surg..

[3] Zozulya S., Echeverri F., Nguyen T. (2001). The human olfactory receptor repertoire. Genome Biol..

[4] Walker H.K., Walker H.K., Hall W.D., Hurst J.W. (1990). Cranial Nerve I: The Olfactory Nerve. Clinical Methods: The History, Physical, and Laboratory Examinations.

[5] Sarafoleanu C., Mella C., Georgescu M., Perederco C. (2009). The importance of the olfactory sense in the human behavior and evolution. J. Med. Life.

[6] Morrison E.E., Costanzo R.M. (1990). Morphology of the human olfactory epithelium. J. Comp. Neurol..

[7] Moran D.T., Rowley J.C., Jafek B.W. (1982). Electron microscopy of human olfactory epithelium reveals a new cell type: The microvillar cell. Brain Res..

[8] Amoore J.E. (1963). Steriochemical theory of olfaction. Nature.

[9] Sell C.S. (2006). On the unpredictability of odor. Angew. Chem. Int. Ed. Engl..

[10] Brookes J.C., Horsfield A.P., Stoneham A.M. (2012). The swipe card model of odorant recognition. Sensors.

[11] Dyson G. (1938). The Scientific Basis of Odour. Chem. Ind..

[12] Wright R.H. (1977). Odor and molecular vibration: Neural coding of olfactory information. J. Theor. Biol..

[13] Turin L. (1996). A spectroscopic mechanism for primary olfactory reception. Chem Senses..

[14] Khemis I., Mechi N., Ben Lamine A. (2018). Stereochemical study of mouse muscone receptor MOR215-1 and vibrational theory based on statistical physics formalism. Prog.Biophys. Mol. Biol..

[15] Gane S., Georganakis D., Maniati K., Vamvakias M., Ragoussis N., Skoulakis E.M., Turin L. (2013). Molecular vibration-sensing component in human olfaction. PLoSONE.

[16] Reese A., List N.H., Kongsted J., Solov’yov I.A. (2016). How Far Does a Receptor Influence Vibrational Properties of an Odorant?. PLoS ONE.

[17] Ngabonziza P., Wang Y., van Aken P., Maier J., Mannhart J. (2021). Inelastic Electron Tunneling Spectroscopy at High-Temperatures. Adv. Mater..

[18] Hoehn R.D., Nichols D., Neven H., Kais S. (2015). Neuroreceptor activation by vibration-assisted tunneling. Sci. Rep..

[19] Tirandaz A., TaherGhahramani F., Salari V. (2017). Validity Examination of the Dissipative Quantum Model of Olfaction. Sci. Rep..

[20] Liza N., Blair E. (2019). An explicit electron-vibron model for olfactory inelastic electron transfer spectroscopy. J. Appl. Phys..

[21] Pandey N., Pal D., Saha D., Ganguly S. (2021). Vibration-based biomimetic odor classification. Sci. Rep..

[22] Block E., Jang S., Matsunami H., Batista V.S., Zhuang H. (2015). Reply to Turin et al.: Vibrational theory of olfaction is implausible. Proc. Natl. Acad. Sci. USA.

[23] Hoehn R.D., Nichols D.E., McCorvy J.D., Neven H., Kais S. (2017). Experimental evaluation of the generalized vibrational theory of G protein-coupled receptor activation. Proc. Natl. Acad. Sci. USA.

[24] Olender T., Lancet D., Nebert D.W. (2008). Update on the olfactory receptor (OR) gene superfamily. Hum. Genom..

[25] Trimmer C., Keller A., Murphy N.R., Snyder L.L., Willer J.R., Nagai M.H., Katsanis N., Vosshall B., Matsunami B.H., Mainland J.D. (2019). Genetic variation across the human olfactory receptor repertoire alters odor perception. Proc. Natl. Acad. Sci. USA.

[26] Huang W., Tang M., Hong C., Lee S. (2019). Investigation of bond oscillation assisted olfactory perception by exciting the molecular chemical bonds using specific IR wavelengths. AIP Adv..

[27] Malnic B., Godfrey P.A., Buck L.B. (2004). The human olfactory receptor gene family. Proc. Natl. Acad. Sci. USA.

[28] Genva M., KenneKemene T., Deleu M., Lins L., Fauconnier M.L. (2019). Is It Possible to Predict the Odor of a Molecule on the Basis of its Structure?. Int. J. Mol. Sci..

[29] Bushdid C., Magnasco M.O., Vosshall L.B., Keller A. (2014). Humans can discriminate more than 1 trillion olfactory stimuli. Science.

[30] Smear M., Resulaj A., Zhang J., Bozza T., Rinberg D. (2013). Multiple perceptible signals from a single olfactory glomerulus. Nat. Neurosci..

[31] Malnic B., Hirono J., Sato T., Buck L.B. (1999). Combinatorial receptor codes for odors. Cell.

[32] Haddad R., Lanjuin A., Madisen L., Zeng H., Murthy V.N., Uchida N. (2013). Olfactory cortical neurons read out a relative time code in the olfactory bulb. Nat. Neurosci..

[33] Omary M., Patterson H. (1999). Luminescence, Theory.

[34] Salas Redondo C., Kleine P., Roszeitis K., Achenbach T., Kroll M., Thomschke M., Reineke S. (2017). Interplay of Fluorescence and Phosphorescence in Organic Biluminescent Emitters. J. Phys. Chem. C Nanomater. Interfaces.

[35] Hipps K.W. (2012). Tunneling spectroscopy of organic monolayers and single molecules. Top. Curr. Chem..

[36] Bigourdan F., Hugonin J.P., Marquier F., Sauvan C., Greffet J.J. (2016). Nanoantenna for Electrical Generation of Surface Plasmon Polaritons. Phys. Rev. Lett..

[37] Zhu Y., Cui L., Abbasi M., Natelson D. (2022). Tuning Light Emission Crossovers in Atomic-Scale Aluminum Plasmonic Tunnel Junctions. NanoLett..

[38] Lebedev D.V., Shkoldin V.A., Mozharov A.M., Larin A.O., Permyakov D.V., Samusev A.K., Petukhov A.E., Golubok A.O., Arkhipov A.V., Mukhin I.S. (2022). Nanoscale Electrically Driven Light Source Based on Hybrid Semiconductor/Metal Nanoantenna. J. Phys. Chem.Lett..

[39] Ahmadivand A. (2021). Electrically Excited Plasmonic Ultraviolet Light Sources. Small.

[40] Parzefall M., Novotny L. (2019). Optical antennas driven by quantum tunneling: A key issues review. Rep.Prog. Phys..

[41] Schaeverbeke Q., Avriller R., Frederiksen T., Pistolesi F. (2019). Single-Photon Emission Mediated by Single-Electron Tunneling in PlasmonicNanojunctions. Phys. Rev. Lett..

[42] Kishen S., Tapar J., Emani N.K. (2022). Tunable directional emission from electrically driven nano-strip metal-insulator-metal tunnel junctions. Nanoscale Adv..

[43] Puchert R.P., Hofmann F.J., Angerer H.S., Vogelsang J., Bange S., Lupton J.M. (2021). Linearly Polarized Electroluminescence from MoS2 Monolayers Deposited on Metal Nanoparticles: Toward Tunable Room-Temperature Single-Photon Sources. Small.

[44] Wang Z., Kalathingal V., Hoang T.X., Chu H.S., Nijhuis C.A. (2021). Optical Anisotropy in van der Waals materials: Impact on Direct Excitation of Plasmons and Photons by Quantum Tunneling. Light Sci. Appl..

[45] Kuzmina A., Parzefall M., Back P., Taniguchi T., Watanabe K., Jain A., Novotny L. (2021). Resonant Light Emission from Graphene/Hexagonal Boron Nitride/Graphene Tunnel Junctions. NanoLett..

[46] Schultz J.F., Li S., Jiang S., Jiang N. (2020). Optical scanning tunneling microscopy based chemical imaging and spectroscopy. J. Phys.Condens. Matter.

[47] Kröger J., Doppagne B., Scheurer F., Schull G. (2018). Fano Description of Single-Hydrocarbon Fluorescence Excited by a Scanning Tunneling Microscope. Nano Lett..

[48] Zhang R., Zhang Y., Dong Z.C., Jiang S., Zhang C., Chen L.G., Zhang L., Liao Y., Aizpurua J., Luo Y. (2013). Chemical mapping of a single molecule by plasmon-enhanced Raman scattering. Nature.

[49] Leon C.C., Rosławska A., Grewal A., Gunnarsson O., Kuhnke K., Kern K. (2019). Photon superbunching from a generic tunnel junction. Sci. Adv..

[50] Joung J.F., Han M., Jeong M., Park S. (2020). Experimental database of optical properties of organic compounds. Sci. Data.

[51] Ye Z.R., Huang I.S., Chan Y.T., Li Z.J., Liao C.C., Tsai H.R., Hsieh M.C., Chang C.C., Tsai M.K. (2020). Predicting the emission wavelength of organic molecules using a combinatorial QSAR and machine learning approach. RSC Adv..

[52] Kazmin R., Rose A., Szczepek M., Elgeti M., Ritter E., Piechnick R., Hofmann K.P., Scheerer P., Hildebrand P.W., Bartl F.J. (2015). The Activation Pathway of Human Rhodopsin in Comparison to Bovine Rhodopsin. J. Biol. Chem..

[53] Hu G.M., Mai T.L., Chen C.M. (2017). Visualizing the GPCR Network: Classification and Evolution. Sci. Rep..

[54] Dryer L., Berghard A. (1999). Odorant receptors: A plethora of G-protein-coupled receptors. Trends Pharmacol. Sci..

[55] Genovese F., Reisert J., Kefalov V.J. (2021). Sensory Transduction in Photoreceptors and Olfactory Sensory Neurons: Common Features and Distinct Characteristics. Front. Cell. Neurosci..

[56] Buck L., Axel R. (1991). A novel multigene family may encode odorant receptors: A molecular basis for odor recognition. Cell.

[57] Fleischer J., Breer H., Strotmann J. (2009). Mammalian olfactory receptors. Front. Cell. Neurosci..

[58] Ballesteros J.A., Shi L., Javitch J.A. (2001). Structural mimicry in G protein-coupled receptors: Implications of the high-resolution structure of rhodopsin for structure-function analysis of rhodopsin-like receptors. Mol. Pharmacol..

[59] Behrens M., Briand L., de March C., Matsunami H., Yamashita A., Meyerhof W., Weyand S. (2018). Structure–Function Relationships of Olfactory and Taste Receptors. Chem. Senses.

[60] Boens N., Qin W., Basarić N., Hofkens J., Ameloot M., Pouget J., Lefèvre J.P., Valeur B., Gratton E., van de Ven M. (2007). Fluorescence lifetime standards for time and frequency domain fluorescence spectroscopy. Anal. Chem..

[61] Fron E., Van der Auweraer M., Moeyaert B., Michiels J., Mizuno H., Hofkens J., Adam V. (2013). Revealing the excited-state dynamics of the fluorescent protein Dendra2. J. Phys. Chem. B.

[62] Canty L., Hariharan S., Liu Q., Haney S.A., Andrews D.W. (2018). Peak emission wavelength and fluorescence lifetime are coupled in far-red, GFP-like fluorescent proteins. PLoS ONE.

[63] Han H., Zhang Z., Weng X., Liu J., Guan X., Zhang K., Li G. (2013). Development of a fast radiation detector based on barium fluoride scintillation crystal. Rev. Sci. Instrum..

[64] Yanagida T. (2018). Inorganic scintillating materials and scintillation detectors. Proc. Jpn. Acad. Ser. B Phys. Biol. Sci..

[65] Schatz G.H., Brock H., Holzwarth A.R. (1987). Picosecond kinetics of fluorescence and absorbance changes in photosystem II particles excited at low photon density. Proc. Natl. Acad. Sci. USA.

[66] Resnati D., Rech I., Geraci A. (2008). High-linearity analog-to-digital acquisition board for photon-timing applications. Rev. Sci.Instrum..

[67] Berezin M.Y., Achilefu S. (2010). Fluorescence lifetime measurements and biological imaging. Chem Rev..

[68] Mukherjee S., Thomas C., Wilson R., Simmerman E., Hung S.T., Jimenez R. (2022). Characterizing dark state kinetics and single molecule fluorescence of FusionRed and FusionRed-MQ at low irradiances. Phys. Chem. Chem. Phys..

[69] Bhandawat V., Reisert J., Yau K.W. (2005). Elementary response of olfactory receptor neurons to odorants. Science.

[70] Caplette L., Ince R.A., Jerbi K., Gosselin F. (2020). Disentangling presentation and processing times in the brain. Neuro Image.

[71] Itoh K., Konoike N., Nejime M., Iwaoki H., Igarashi H., Hirata S., Nakamura K. (2022). Cerebral cortical processing time is elongated in human brain evolution. Sci. Rep..

[72] Williams L., Linzen T., Poeppel D., Marantz A. (2018). In Spoken Word Recognition, the Future Predicts the Past. J. Neurosci..

[73] Williams L., King J.R., Marantz APoeppel D. (2022). Neural dynamics of phoneme sequences reveal position-invariant code for content and order. Nat. Commun..

[74] Palczewski K. (2012). Chemistry and Biology of Vision. J. Biol. Chem..

[75] Zhang S., Cui Y., Li X., Sun Y., Wang Z. (2022). Multiphonon processes of the inelastic electron transfer in olfaction. Phys. Chem. Chem. Phys..

[76] Jorgensen W., Gao J. (1988). Cis-trans energy difference for the peptide bond in the gas phase and in aqueous solution. J. Am. Chem. Soc..

[77] Cembran A., Bernardi F., Garavelli M., Gagliardi L., Orlandi G. (2004). On the mechanism of the cis-trans isomerization in the lowest electronic states of azobenzene: S0, S1, and T1. J. Am. Chem. Soc..

[78] Li Y.S., Escobar L., Zhu Y.J., Cohen Y., Ballester P., Rebek J., Yu Y. (2019). Relative hydrophilicities of cis and trans formamides. Proc. Natl. Acad. Sci. USA.

[79] Najafloo R., Majidi J., Asghari A., Aleemardani M., Kamrava S.K., Simorgh S., Seifalian A., Bagher Z., Seifalian A.M. (2021). Mechanism of Anosmia Caused by Symptoms of COVID-19 and Emerging Treatments. ACS Chem. Neurosci..

[80] Brann D.H., Tsukahara T., Weinreb C., Lipovsek M., Van den Berge K., Gong B., Chance R., Macaulay I.C., Chou H.J., Fletcher R.B. (2020). Non-neuronal expression of SARS-CoV-2 entry genes in the olfactory system suggests mechanisms underlying COVID-19-associated anosmia. Sci. Adv..

[81] Mutiawati E., Fahriani M., Mamada S.S., Fajar J.K., Frediansyah A., Maliga H.A., Ilmawan M., Emran T.B., Ophinni Y., Ichsan I. (2021). Anosmia and dysgeusia in SARS-CoV-2 infection: Incidence and effects on COVID-19 severity and mortality, and the possible pathobiology mechanisms—A systematic review and meta-analysis. F1000Research.

[82] Karimian A., Behjati M., Karimian M. (2022). Molecular mechanisms involved in anosmia induced by SARS-CoV-2, with a focus on the transmembrane serine protease TMPRSS2. Arch. Virol..

[83] Butowt R., von Bartheld C.S. (2021). Anosmia in COVID-19: Underlying Mechanisms and Assessment of an Olfactory Route to Brain Infection. Neuroscientist.

[84] Patel S., Tutchenko L. (2019). The refractive index of the human cornea: A review. Contact Lens Anterior Eye.

[85] Zhang Y., Pan Y., Matsunami H., Zhuang H. (2017). Live-cell Measurement of Odorant Receptor Activation Using a Real-time cAMP Assay. J. Vis. Exp..

[86] De March C.A., Fukutani Y., Vihani A., Kida H., Matsunami H. (2019). Real-time In Vitro Monitoring of Odorant Receptor Activation by an Odorant in the Vapor Phase. J. Vis. Exp..

